# Paricalcitol supplementation during the first year after kidney transplantation does not affect calcification propensity score

**DOI:** 10.1186/s12882-018-1000-8

**Published:** 2018-08-22

**Authors:** Amin Ussif, Hege Pihlstrøm, Andreas Pasch, Hallvard Holdaas, Anders Hartmann, Knut Smerud, Anders Åsberg

**Affiliations:** 1 Department of Transplantation Medicine, Oslo University Hospital Rikshospitalet, P.O. Box 4950, Nydalen, 0424 Oslo, Norway; 2 Calciscon AG, Bern, Switzerland; 3 Institute of Clinical Medicine, Faculty of Medicine, University of Oslo, Oslo, Norway; 4 Smerud Medical Research International AS, Oslo, Norway; 5 The Norwegian Renal Registry, Oslo University Hospital Rikshospitalet, Oslo, Norway; 6 Department of Pharmaceutical Biosciences, School of Pharmacy, University of Oslo, Oslo, Norway

**Keywords:** Paricalcitol, Calcification propensity score, Randomized controlled trial, Renal transplantation

## Abstract

**Background:**

Cardiovascular complications are common in kidney transplant patients and calcification propensity of blood, measured as T_50_, is associated with cardiovascular outcomes. Paricalcitol supplementation affects calcium/phosphate homeostasis and may affect calcification propensity. To assess this hypothesis we measured T_50_ in kidney transplant recipients participating in a randomized study comparing paricalcitol versus no treatment during the first year after kidney transplantation.

**Methods:**

Stored serum samples from 76 kidney transplant recipients (paricalcitol *n* = 37, no treatment *n* = 39) were analyzed. Analyses were performed at inclusion (8 weeks after transplantation) and repeated one year after transplantation.

**Results:**

There were no statistically significant differences in T_50_ between the paricalcitol and placebo groups, neither at baseline (*p* = 0.56) nor at 1 year (*p* = 0.61). Also, there were no significant changes in T_50_ over time in either group or when pooling all data (*p* <  0.20). In multivariate regression analysis, out of 16 potentially relevant covariates, comprising clinical and biochemical parameters, only plasma PTH and T_50_ at baseline were significantly correlated to T_50_ after one year. (*p* <  0.03 and *p* < 0.01, respectively).

**Conclusions:**

Calcium propensity measured as T_50_ score remained unchanged with paricalcitol treatment in kidney transplant recipients, and was not changed over time during the study period of one year.

**Trial registration:**

ClinicalTrials.gov, NCT01694160, registered 23 September 2012.

## Background

Patients with chronic kidney disease have increased risk of cardiovascular complications and impaired survival [1]. Vascular calcification is a prominent feature in chronic kidney disease (CKD) patients that is also associated with cardiovascular morbidity and mortality [2, 3]. Restoration of kidney function following kidney transplantation seems to alleviate the burden of cardiovascular complications, but the risk remains substantially increased compared with the general population [4, 5].

Calcium/phosphate homeostasis undergoes substantial changes following transplantation. There have been some indications that low 25-hydroxyvitamin D (25-OH)D may be associated with proteinuria and fibrosis in renal transplant recipients [6, 7]. Paricalcitol (19-nor-1,25-dihydroxyvitamin D2) is a synthetic, selective third generation vitamin D receptor agonist associated with low risk of hypercalcaemia [8, 9]. In CKD, paricalcitol effectively and safely suppresses PTH and may reduce proteinuria. Similar effects, although less established, have been suggested in renal transplant recipients [10].

We therefore recently performed a randomized controlled trial (RCT) comparing paricalcitol and no treatment to assess effects on albuminuria, inflammation related gene expression profiles and fibrosis in protocol biopsies [10]. The study did not reveal any effects on these parameters, but plasma parathyroid hormone (PTH) levels were as expected reduced in the active treatment group. We speculated that the effects on PTH and calcium/phosphate might influence the calcification propensity of the patients, which can be measured in serum by a novel test [11]. The test readout (T_50_) reflects the functionality of a physiologic system, the system of humoral calcification control, by measuring the time of transformation of soluble primary calciprotein particles (CPPs) to secondary, crystalline (hydroxyapatite-containing) CPPs during stimulation of calcification in vitro. T_50_ is the time to half maximal transition to insoluble crystalline CPPs and a lower T_50_ signifies increased tendency for calcification. It has recently been found that T_50_ is a strong and independent risk factor for adverse outcomes including cardiovascular death both in CKD [12, 13] and kidney transplant recipients [14, 15].

In the present study the primary aim was to reveal any possible effect of paricalcitol supplementation on T_50_ scores during the first year after transplantation.

## Methods

Study participants were recruited in the period of Jan 2013 to Feb 2014 from the National Transplant Centre at Oslo University Hospital, Rikshospitalet, Oslo, Norway. The original study was an open-label, randomized controlled trial comparing paricalcitol treatment to standard of care treatment in de-novo renal transplant recipients. Recipients are routinely followed for 8–10 weeks after engraftment and return for a routine one-year surveillance follow-up. The random allocation sequence was generated by an independent statistician at the monitoring facility, using computer-generated block-randomization with nonfixed block size. The principal investigator (PI) performed the opening of sealed envelopes and informed the participants of their group assignment. Treatment allocation remained undisclosed to the staff performing laboratory measurements [10]. In short, patients were invited to participate in the study in a stable phase 7–8 weeks after transplantation.

Patients receiving standard calcineurin inhibitor based triple immunosuppression with eGFR > 30 ml/min/1.73 m^2^ with normocalcemia were considered for inclusion. Previous parathyroidectomy, treatment with vitamin D or calcimimetics were the main exclusion criteria. The patients were randomised to receive open label treatment with oral paricalcitol (Zemplar® (Abbvie) 2 μg/day) or no treatment. Serum samples were obtained at time of randomization and at one year after transplantation and stored at minus 70 °C. The analysis of T_50_ scores was performed in one batch using laser detection of fully calcified particles as described in detail elsewhere [11].

In short, analyses were performed in triplicates in on 384-well plates. Patient serum (40 uL) was exposed to high and supersaturated concentrations of calcium (35 uL) and phosphate (25 uL). The transformation step was then monitored at 37 °C using time-resolved nephelometry (bmg labtech, Ortenberg, Germany). Nonlinear regression curves were calculated, allowing the determination of the one-half maximal transition time (T50).

Adequate samples for analysis of T_50_ were obtained from all patients in the treatment arm (*n* = 37) and all except one in the control group (*n* = 39). Demographic and transplant related data of the patients in the two groups are shown in Table 1.

**Table 1:** Demographics and transplant characteristics, mean (SD), at baseline (8 weeks − 1 year after renal transplantation) -

Variables	Paricalcitol *N* = 37	Paricalcitol *N* = 37	No treatment *N* = 39	No treatment *N* = 39
Time point post Tx	8 weeks	1 year	8 weeks	1 year
Age (years)	56.0 (13.4)	NA	54.2 (12.1)	NA
Albumin (g/L)	42.4 (2.4)	43.3 (2.5)	41.7 (2.5)	43.3 (2.7)
ALP (U/L)	61.4 (21.7)	58.4 (20.5)	70.2 (28.0)	71.4 (33.4)
BMI (kg/m^2^)	26.1 (3.2)	NA	25.5 (3.9)	NA
Calcium, total (mmol/L)	2.4 (0.1)	2.4 (0.1)	2.4 (0.1)	2.4 (0.1)
Creatinine (μmol/L)	114.4(24.5)	110.3 (26.7)	120.9 (29.6)	114.6 (27.5)
Phosphate (mmol/L)	0.9 (0.2)	0.9 (0.1)	0.9 (0.3)	0.9 (0.1)
PTH (pmol/L)	11.5 (5.7)	7.9 (4.8)	11.4 (5.3)	9.9 (3.8)
T_50_ (min)	311 (35)	319 (34.5)	319 (34)	323 (35.6)
HLA mismatch	3.1 (1.3)	NA	3.0 (1.4)	NA
Gender (% male)	27 (73)	NA	33 (84.6)	NA
Smoking (% Yes)	5 (13.5)	NA	5 (12.8)	NA
Living donor (% Yes)	10 (27.0)	NA	13 (33.3)	NA
Predialytic transplantation (% Yes)	13 (31.1)	NA	12 (30.8)	NA
Pretransplant diabetes (% Yes)	7 (18.9)	NA	6 (15.4)	NA

NA denotes not available. For clarity all figures are reported to two decimals

Abbreviations: *ALP* alkaline phosphatase, *BMI* body mass index, *PTH* parathyroid hormone, T_50_; time to 50% transfer to crystalline particles, *HLA* human leukocyte antigen

### Statistical methods

Baseline summary data of the total study population (*n* = 76) was calculated using descriptive analysis tool. To identify possible confounders and predictors for the subsequent analysis Pearson correlation analysis was also conducted (results not reported). Both univariate and multivariate linear regression models were employed in our analysis. Treatment, demographic and biochemical variables were included in the linear regression models. The inclusion criteria for the multiple regression models were based on clinical and biological plausibility. All analysis was performed using IBM SPSS Statistics version 24, release 24.0.0.1 and plots were done using MS Excel 2010.

The study was performed in compliance with GCP and the Helsinki declaration and approved by Norwegian Medicines Agency and the Regional Ethics committee of Health region South-East and registered on www.clinicaltrial.gov (NCT01694160 (2012/107D)).

## Results

Demographic, clinical and biochemical characteristics of the population under study are summarized in Table 1. No clinically significant differences were observed between the groups at baseline. In particular, the means of T_50_ scores did not differ significantly at baseline; 311 ± 35 min in the paricalcitol 316 ± 48 min in the no treatment group, respectively (*p* = 0.56). Both groups were predominantly male (78.9%) with mean age of 55 ± 12.7 years old and plasma PTH of 11.5 ± 5.5 pmol/L. In total 31% of patients were predialytic at time of transplantation, and 30% received organs from living donors. Only 13% were current smokers, while 45% were previous smokers. Details of the baseline characteristics are discussed in Pihlstrøm et al. [10].

**Fig. 1:**
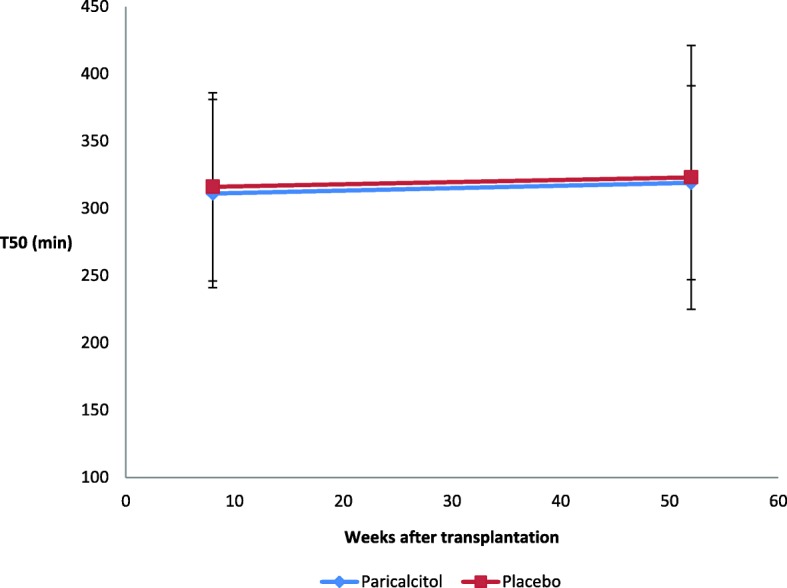
Mean T_50_ (min) after renal transplantation (paricalcitol vs placebo)

Figure 1 is a graphical display of the means ±2 SDs of T_50_ scores for the paricalcitol vs no treatment group at baseline (i.e. eight weeks after transplantation) and after one year. The trend lines between the two measurement points show no significant change over time and no difference between groups based on paired t-test, ANOVA and a simple linear regression on the categorical variables (data not shown).

Table 2 shows univariate and multivariate linear regression analyses. The regression constant is not reported here for brevity. In univariate analysis, only T_50_ score at baseline (*p* < 0.01) is significantly associated with T_50_ score at one year, while baseline plasma PTH (*p* = 0.09), pretransplant dialysis (*p* = 0.09) and living donor (*p* = 0.08) are not significantly associated with T_50_ score at study end. The statistical significant variables in the multivariate analysis were T_50_ score at baseline (*p* < 0.01) and baseline PTH (*p* = 0.031). From the standardized (beta) coefficients the T_50_ score at baseline (beta = 0.43) and baseline PTH (beta = − 0.25) are the two most important predictors. The overall multivariate regression model (F = 1.48, *p* = 0.14) is not statistically significant. Goodness of fit (R^2^) equals 0.29; hence our model explains only about 30% of that data.

**Table 2:** Uni- and multivariate linear regression analysis of predictors of T_50_ at one year

	Univariate		Multivariate	
Variable	β (t-values)*	*p*-value	β (t-values)*	*p*-value
Age (years)	−0.15 (−0.48)	0.64	0.22 (0.64)	0.53
Gender (% male)	0.079 (0.01)	0.99	−6.30 (− 0.48)	0.63
BMI (kg/m^2^)	0.84 (0.74)	0.46	0.69 (0.57)	0.57
Smoking	0.65 (0.08)	0.94	−3.12 (−0.25)	0.80
HLA mismatch	1.06 (0.36)	0.72	0.81 (0.25)	0.80
Living donor	15.23 (1.77)	0.08	−2.83 (−0.26)	0.79
Predialytic transplantation	11.13 (1.31)	0.19	11.12 (1.15)	0.25
Pretransplant diabetes	−17.65 (−1.68)	0.107	−5.02 (−0.39)	0.70
Treatment	−4.12 (−0.51)	0.61	0.37 (0.04)	0.97
T_50_ baseline (min)	0.36 (4.12)	< 0.01	0.37 (3.44)	< 0.01
Albumin (g/L)	1.03 (0.64)	0.64	0.15 (0.08)	0.94
ALP (U/L)	0.02 (0.12)	0.90	0.18 (0.95)	0.35
Calcium (mmol/L)	3.48 (0.07)	0.93	−6.11 (−0.12)	0.90
Creatinine (μmol/L)	0.01 (0.06)	0.95	−0.01 (− 0.04)	0.97
Phosphate (mmol/L)	−4.79 (−0.30)	0.76	5.07 (0.28)	0.78
PTH (pmol/L)	−1.61 (−2.24)	0.09	−1.70 (−2.21)	0.03

Abbreviations: *ALP* alkaline phosphatase, *BMI* body mass index, *PTH* parathyroid hormone, T_50_; time to 50% transfer to crystalline particles, *HLA* human leukocyte antigen

Estimates are reported together with their t-values in parenthesis and *p*-values are in separate columns. *t-value at 95% cutoff 1.96

## Discussion

We studied the potential effects of paricalcitol supplementation during the first year after kidney transplantation on calcification propensity score in his paper. Available data show that treatment with paricalcitol did not significantly affect T_50_. Calcification propensity measured as serum T_50_ scores at baseline and after one year of treatment with paricalcitol in a RCT was analyzed in the present study. The novel finding in the present analysis was that all analyses demonstrated that paricalcitol treatment during the first year after transplantation did not influence T_50_ score.

In addition to this important but negative finding, our qualitative analysis shows a flat trend in the T_50_ score, which is similar to earlier results reported for T_50_ in kidney transplant recipients [15]. In another study, assessing the effect of ibandronate over time, T_50_ scores increased substantially during the first few weeks after transplantation, but then remained unchanged over the first year regardless of treatment allocation [16].

In the present original study, paricalcitol treatment was significant associated with a reduction in serum PTH [10]. However, the reduction in plasma PTH in the treatment arm did not associate with a change in T_50_ score. On the contrary, plasma PTH at baseline was the only clinical covariate showing independent associations with T_50_ score at one year. This may indicate that elevated PTH early after transplantation reflects long standing effects associated with hyperparathyroidism that may be more important than changes following normalization of kidney function during the first posttransplant year. There are several factors that influence T50 scores that are not account for in this post-hoc study. Plasma magnesium and bicarbonate are significant contributors to factors influencing T_50_ scores, unfortunately, these were not measured. Magnesium levels are generally low in kidney transplant recipients, especially in the early phase after transplantation. The bicarbonate concentration is first of all dependent on kidney function. Plasma magnesium may normalize within the first year whereas bicarbonate usually remains within normal values as long as the kidney function remains stable. We did not find any change in T_50_ over the first year that could beexplained by variations in these parameters. In any case there is no reason to believe that magnesium and bicarbonate concentrations would change differently with or without paricalcitol treatment.

### Strengths and limitations

The current investigation is a *post-hoc* analysis that was not predetermined, which is an obvious weakness and it should be interpreted accordingly. Also the lack of effect of paricalcitol beyond lowering of PTH in the primary study may be considered a downside for effect studies on other parameters like T_50_. However, the study was indeed a properly performed RCT.

## Conclusions

To summarize, treatment with paricalcitol did not have any effect on T_50_ in renal transplant recipients as compared with controls without treatment. Also there were no differences in T_50_ scores at baseline or at one year in either group. Baseline PTH levels were significantly associated with T_50_ score at one year. Further studies are warranted to assess the therapeutic efficacy of interventions such as magnesium on clinical outcome measures.

## Abbreviations

(25-OH)D: 25-hydroxyvitamin D
ANOVA: Analysis of variance
CKD: Chronic kidney disease
CNI: Calcineurin inhibitor
CPPs: Primary calciprotein particles
eGFR: Estimated glomerular filtration rate
PTH: Parathyroid hormone
RCT: Randomized controlled trial
SD: Standard deviation
